# Training Sri Lankan public health midwives on intimate partner violence: a pre- and post-intervention study

**DOI:** 10.1186/s12889-015-1674-9

**Published:** 2015-04-07

**Authors:** Achini Chinthika Jayatilleke, Kayoko Yoshikawa, Junko Yasuoka, Krishna C Poudel, Nilani Fernando, Achala Upendra Jayatilleke, Masamine Jimba

**Affiliations:** Department of Community and Global Health, Graduate School of Medicine, The University of Tokyo, Tokyo, Japan; Department of Health Promotion and Policy, School of Public Health and Health Sciences, University of Massachusetts Amherst, Amherst, MA USA; Regional Director of Health Services Office, Ministry of Health, Kandy, Sri Lanka; Postgraduate Institute of Medicine, University of Colombo, Colombo, Sri Lanka

**Keywords:** Intimate partner violence, Sri Lanka, Public health midwives, Training

## Abstract

**Background:**

In many developing countries, intimate partner violence (IPV) training is not available for health providers. As a pioneer among developing countries, in 2009, the Sri Lankan Ministry of Health trained a group of community health providers known as public health midwives (PHMs) on IPV. We evaluated that training program’s efficacy in improving PHMs’ identification and management of IPV sufferers in Kandy, Sri Lanka.

**Methods:**

We conducted this study from August 2009 to September 2010. We used a self-administered structured questionnaire to examine the following variables among 408 PHMs: self-reported IPV practices, IPV knowledge, perceived barriers, perceived responsibility, and self-confidence in identifying and assisting IPV sufferers. We used McNemar’s test to compare PHMs’ pre- and post-intervention IPV practices. Using the Wilcoxon signed-rank test, we compared PHMs’ pre-and post-intervention IPV knowledge, as well as their perceived barriers, responsibility, and self-confidence scores.

**Results:**

The IPV training program improved PHMs’ IPV practices significantly. Six months after the intervention, 98.5% (n = 402) of the 408 PHMs identified at least one IPV sufferer in the previous three months, compared to 73.3% (n = 299) in the pre-intervention (*p* < 0.001). At post-intervention, 96.5% (n = 387) of the PHMs discussed IPV with identified sufferers and suggested solutions; only 67.3% (n = 201) did so at the pre-intervention (*p* < 0.001). In addition, after the intervention, there were significant increases (*p* < 0.001) in the median total scores of PHMs’ IPV knowledge (0.62 vs. 0.88), perceived responsibility (3.20 vs. 4.60), and self-confidence (1.81 vs. 2.75). PHMs’ perceived barriers decreased from 2.43 to 1.14 (*p* < 0.001).

**Conclusions:**

An IPV training program for PHMs improved identification and assistance of IPV sufferers in Kandy, Sri Lanka. This training program has the potential to improve PHMs’ skills in preventing IPV and supporting sufferers in other regions of Sri Lanka. Other developing countries might learn lessons from Sri Lanka’s IPV training.

**Electronic supplementary material:**

The online version of this article (doi:10.1186/s12889-015-1674-9) contains supplementary material, which is available to authorized users.

## Background

Intimate partner violence (IPV) is an important public health problem associated with several adverse health consequences ranging from minor bruises to severe depression and suicide [1-3]. Sufferers of IPV frequently visit health facilities with IPV-related health problems, but rarely disclose their experiences with IPV [4-6]. As a result, in most instances, IPV is not recognized and properly addressed by health providers. This situation is common in developing countries [4,6].

Several barriers might prevent health providers from recognizing IPV, including lack of IPV training, lack of time, fear of offending women, lack of self-confidence in IPV identification, and lack of skills in responding to IPV disclosures [6-8]. Of these, lack of IPV training is an important barrier preventing health providers from recognizing IPV [8,9]. This lack of training might contribute to health providers’ lack of skills and confidence in identifying and responding to IPV [4,6].

IPV training programs have significantly improved health providers’ recognition and management of IPV sufferers in developed countries [8-12]. However, in many developing countries, IPV training is not available for health providers, and health providers rarely recognize or support IPV sufferers in health care settings [4,6]. When sufferers disclose IPV, health providers hesitate to get involved and are reluctant to help them because IPV is not usually considered a health concern [4,7].

Providing IPV training to health providers might be particularly challenging in developing countries [4,6]. In these countries, IPV is often justified as a normal part of a marital relationship and as a private family matter [1,3,6]. Health workers can provide only a limited number of IPV services; shelters for sufferers are limited and social services provide inadequate support [4,6]. Therefore, in developing countries, health providers need to learn how to use the available resources and provide culturally sensitive IPV services by training.

In Sri Lanka, one of every three married women experience IPV [13-16]. In a study conducted in the Central Province of Sri Lanka, 36% of 624 wives had experienced at least one episode of physical, psychological, or sexual violence from their husbands during their lifetime [13]. Another study, conducted with 728 wives in the Western Province, reported that the lifetime prevalence of physical IPV was 34% [14]. However, only a limited number of IPV sufferers seek health care in Sri Lanka [14,17]. Therefore, it is important to identify IPV sufferers in the community, as identification might prevent these women from developing serious health problems associated with IPV.

In Sri Lanka, public health midwives (PHMs) are the most common community health providers working closely with women in the community [18-20]. Their tasks include providing family planning services, antenatal care, postnatal care, and child immunization free of charge to all women and children in their work area [18,20]. Their dedicated service has helped Sri Lanka achieve the best reproductive health indices in the South Asian region. For example, the maternal mortality rate in Sri Lanka was 29 per 100,000 live births in 2013, compared to 190, 190, and 170 in India, Nepal, and Bangladesh, respectively [21]. The Sri Lankan Ministry of Health (MOH) recruits PHMs from the localities in which they are likely to work; females older than 18 who have a minimum of 10 years of formal education are selected for 18 months of midwife training. After the training, one PHM serves a population of approximately 3,000 women across approximately 750 households. PHMs conduct field visits in order to ensure the health and wellbeing of their allocated population. PHMs may visit each household approximately once per month [18,20,22]. As PHMs visit women in their homes, they can observe women in the environment where IPV occurs. As all PHMs are female, they can build trusting relationships with women in the community.

As a pioneer among developing countries, in 2009, the MOH in Sri Lanka introduced an IPV training program to PHMs. This IPV training program aimed to improve PHMs’ identification of IPV and their assistance of identified sufferers [19,20]. It was designed and conducted by the Family Health Bureau of the MOH, and the United Nations Population Fund (UNFPA) provided financial assistance [19,20]. Before introducing the program nationwide, the MOH first piloted the program with PHMs in one district (Kandy) in the Central Province to assess its efficacy for improving PHMs’ IPV practices. In this study, we evaluated the efficacy of the IPV training program to improve PHMs’ identification and management of IPV sufferers in the Kandy district of Sri Lanka.

## Methods

### Setting

We conducted this pre- and post-intervention study in the Kandy district of Sri Lanka between August 2009 and September 2010. Kandy is the second-largest district in Sri Lanka, with a population of 1.4 million. Of that population, 12.1% live in urban areas, 82% live in rural areas, and 5.9% live in tea plantation estate areas [23]. The basic health care unit is called a Medical Officer of Health area; one community physician (a Medical Officer of Health) is responsible for the primary health care services in one area. In each area, 30–35 PHMs provide primary health care services under the supervision of the Medical Officer of Health. For administrative purposes, the MOH refers to the PHMs working in urban and rural areas as “field PHMs.” PHMs working in tea plantation estate areas are called “estate PHMs.” In Kandy, there are 22 Medical Officer of Health areas. During the study period, a total of 495 PHMs (field and estate) provided primary health care services in those 22 areas [19,22].

### Participants

**Figure 1:**
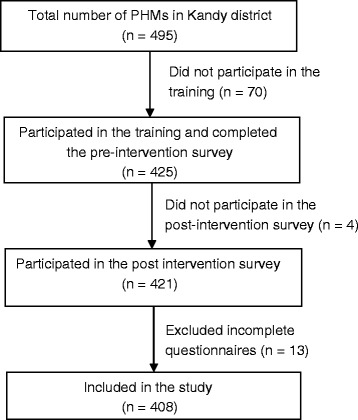
**Participant selection for the pre- and post-intervention surveys.**

As shown in Figure 1, we recruited all the PHMs in Kandy district (n = 495) for our study. Among them, 425 participated in the pre-intervention survey; the other 70 PHMs did not participate due to personal reasons (e.g., illness). For the post-intervention survey, we recruited the PHMs who participated in the pre-intervention survey (n = 425). Among them, four PHMs did not participate due to personal reasons. We also excluded 13 participants whose questionnaires were incomplete. Final analyses evaluated the data of 408 PHMs who had worked in Kandy for more than one year.

### Training program (intervention)

The PHMs’ IPV training program consisted of four days of training. A group of gender-based violence (GBV) experts designed the program according to the international guidelines on training health providers on IPV [24-26]. These experts included community physicians, obstetricians and gynecologists, and psychologists who attended international training and had experience with GBV. They prepared a 60-page, A4-size training manual for trainers and a 52-page, A5-size field handbook for the PHMs. As per international guidelines, those materials were prepared in the PHMs’ local language (Sinhala), matched PHMs’ professional requirements, and aimed to improve PHMs’ practical IPV skills [24-26]. Subsequently, the experts trained five community physicians in Kandy as trainers to conduct the IPV training program with PHMs. Those trainers were all the community physicians in Kandy who held masters or doctoral postgraduate qualifications in public health.

The trainers conducted 11 four-day training programs for the PHMs working in 22 Medical Officer of Health areas. They combined the PHMs of two Medical Officer of Health areas for each training program. The training was done at the office of one of the two Medical Officer of Health areas. Approximately 60–65 PHMs participated in each four-day training program. The contents of the training were as follows: (1) gender roles; (2) the types, acts, and health effects of IPV; (3) the domestic violence (DV) prevention law in Sri Lanka; (4) the available supportive services for IPV sufferers in the country; and (5) how to identify and assist IPV sufferers.

Using role-playing and case reports, trainers discussed how to manage IPV sufferers in different situations, and improved the PHMs’ practical IPV skills. Because IPV is a culturally sensitive issue in Sri Lanka [16,27], trainers requested the PHMs to respect cultural norms when intervening to help sufferers. Furthermore, they stressed the importance of adequate privacy when inquiring about IPV and required the PHMs to keep sufferers’ information confidential. As Sri Lanka has a few IPV referral resources, trainers required PHMs to network with local women’s groups and social services to refer sufferers locally. PHMs could also refer sufferers to the Medical Officers of Health, who provided psychological care for the sufferers and referred them to legal services as required. At the end of the training program, the MOH gave each PHM a field handbook that contained a summary of what they learned during the program.

### Study instrument

We used a self-administered structured questionnaire for data collection. We prepared the questionnaire using the World Health Organization’s (WHO) Multi-country Study on Women’s Health and Domestic Violence against Women [1,2], and existing scales that examined health providers’ perceived barriers [7-11], self-confidence [7,8,11,12], and responsibility [8,12] in identifying and assisting IPV/DV sufferers. We adopted items from those previously validated scales, and prepared our scales to suit the Sri Lankan context.

We assessed IPV knowledge using 16 items. For each item, PHMs could respond *yes*, *no*, or *don’t know*. One mark was given for each correct answer. PHMs’ perceived barriers and responsibility in identifying IPV sufferers were assessed using seven and five items, respectively. For each item, PHMs were asked to respond using a five-point Likert scale ranging from 1 (*strongly disagree*) to 5 (*strongly agree*). PHMs’ self-confidence in assisting IPV sufferers was assessed using eight items. For each item, PHMs were asked to respond using a three-point Likert scale as follows: 1 (*not confident*), 2 (*somewhat confident*), and 3 (*very confident*).

We examined three practices regarding IPV: the identification of at least one new IPV sufferer during the past three months of the study, the discussion of IPV with the identified IPV sufferers and suggested solutions to prevent or reduce further violence, and follow-up with the identified IPV sufferers. We asked PHMs whether they had identified IPV sufferers currently experiencing IPV. We did not ask whether they identified sufferers that had ever experienced IPV, as sufferers might not disclose past IPV to the PHMs and might not have displayed external signs of IPV for the PHMs to identify.

When designing our questionnaire, we used the following definition of IPV: “violence against wives or husbands by their current or former husbands or wives, in the form of physical, psychological, or sexual violence” [1-4]. Living together without being married and pre-marital or extra-marital relationships are not socially acceptable practices in Sri Lanka. Therefore, we did not include IPV by boyfriends or girlfriends. Moreover, because none of the PHMs identified a husband experiencing IPV from his wife, we only reported PHMs’ identification and assistance of wives who experienced IPV.

After developing the questionnaire, two Sri Lankan GBV experts evaluated it for clarity, appropriateness for the Sri Lankan context, and ability to assess the efficacy of the program. We modified the questionnaire using the expert comments and translated it into Sinhala and Tamil, the two official languages of Sri Lanka. We then back-translated the questionnaires into English. Based on the back translations, we made the necessary modifications in the forward-translated questionnaires. For example, the following was an item in the PHMs’ perceived responsibility scale: “Telling a person that the violence can adversely affect his/her health.” In the original scale [8], this item read as “Telling a patient that a particular relationship is harmful to his/her health.” This conveyed a harsh meaning in the forward translation; rather than stressing that IPV is harmful to the person, it stressed that the relationship is harmful to the person. Therefore, we rephrased it to the current form. Further, because PHMs provide services to healthy people and not patients, we rephrased the term “patient” to “person.” These modifications improved the clarity as well as the cultural acceptability of the item.

We pre-tested the study questionnaire with 47 PHMs in a different district (Nuwaraeliya). The Cronbach’s alpha was 0.80, 0.89, and 0.87 for the perceived barriers, perceived responsibility, and self-confidence scales, respectively. The questionnaire showed adequate test-retest reliabilities (>0.80) for all of the domains.

To examine the predictive validity of the PHMs’ perceived barriers, perceived responsibility, and self-confidence scales, we examined the correlations of the PHMs’ IPV knowledge scores with their perceived barriers, responsibility, and self-confidence scores using Spearman’s rank correlation coefficient. As indicated in the Additional file 1, at baseline and at six months follow-up, PHMs’ perceived responsibility and self-confidence scores showed significant positive correlations with IPV knowledge and with each other. The perceived barrier scores negatively correlated with PHMs’ IPV knowledge, self-confidence, and responsibility scores. This finding is consistent with previous studies indicating health providers’ IPV knowledge positively correlates with their self-confidence and perceived responsibility to assist IPV sufferers, and negatively correlates with their perceived barriers [7,8]. Hence, the three scales used in our study carried adequate predictive validity.

### Data collection

We administered the pre-intervention surveys just before commencing each IPV training program. Post-intervention surveys were conducted six months after each pre-intervention survey. To maximize PHMs’ participation in the post-intervention surveys, we administered the questionnaires at their monthly meetings. All PHMs completed the questionnaire within 40 minutes. We used ID numbers to match the PHMs’ pre- and post-intervention responses.

### Data analysis

We used SPSS version 17 statistical software (Chicago, USA) for all of the statistical analyses. Using descriptive statistics, we first summarized participants’ socio-demographic and job characteristics. We then reported the frequencies of PHMs’ pre- and post-intervention IPV practices to identify and assist IPV sufferers. We used McNemar’s test to compare those practices before and after the intervention. Using the Wilcoxon signed-rank test, we compared pre- and post-intervention responses for PHMs’ IPV knowledge, perceived barriers, responsibility, and self-confidence. We used the Wilcoxon signed-rank test because all of these variables had non-normal distributions [28]. We examined the changes in responses to individual items as well as summary scores to assess the efficacy of the training program. We created scores for each domain by summing the scores for items in the domain and dividing by the number of items answered. Scores for IPV knowledge ranged from 0 to 1, scores for the perceived barriers and responsibility scales ranged from 1 to 5, and scores for the self-confidence scale ranged from 1 to 3.

### Ethical considerations

We obtained ethical approval for this study from the Research Ethics Committee of The University of Tokyo, Japan, and the Research Ethics Committee of the University of Peradeniya, Sri Lanka. Permission to conduct the study was also obtained from the Provincial Director of Health Services, Central Province, Sri Lanka. All participants signed an informed consent form before questionnaire administration.

## Results

### Characteristics of the study population

Of all the PHMs included in the study (n = 408), 95.1% were field PHMs, 4.2% were estate PHMs, and 0.7% were supervising PHMs. Of all, 85.8% were married, 11.8% were unmarried, and 2.4% were widows; none of the PHMs were divorced or separated at the time of the study. The median age of the 408 PHMs was 43 years (interquartile range [IQR]: 36.5–51 years), and 62.5% were above 40 years old. The majority of the PHMs (74.3%) had 12 or more years of formal education prior to their enrollment in midwifery schools; the rest had 10 years of formal education. PHMs’ median work duration was 17 years (IQR: 10–23 years), and 70.4% had worked as PHMs for more than 10 years. The work duration as field PHMs was more than 10 years, 5–10 years, and less than 5 years for 63%, 18.6%, and 18.4% of participants, respectively. Some PHMs had worked in hospital settings before commencing work as field PHMs.

### PHMs’ pre- and post-intervention practices to identify and assist IPV sufferers

Table 1 shows the PHMs’ identification of IPV sufferers before the intervention and six months after. Six months after the intervention, 98.5% of the 408 PHMs identified at least one IPV sufferer over the previous three months, while this value was 73.3% pre-intervention. Before the intervention, 53.8% of the PHMs identified IPV sufferers because the relatives or friends of those sufferers disclosed the IPV to the PHMs. However, after the intervention, 46.7% of the PHMs identified IPV based on the sufferers’ disclosures, while just 28.1% identified sufferers based on relatives’ or friends’ disclosures. Importantly, 22.1% identified sufferers based on external signs of possible IPV, such as unusual injuries or depressed mood.

**Table 1:** **Participants’ pre- and post-intervention practices to identify intimate partner violence (IPV) sufferers**

**Variable**	**Pre-intervention**	**Post-intervention**
	**n (%)**	**n (%)**
**Newly identified at least one IPV sufferer during the past three months** ^*^		
Yes	299 (73.3)	402 (98.5)
No	109 (26.7)	6 (1.5)
**Number of IPV sufferers identified during the past three months**		
One	109 (36.4)	50 (12.5)
Two	188 (62.9)	289 (71.9)
Three	2 (0.7)	63 (15.6)
**The ways in which PHMs identified IPV sufferers** ^**^		
Sufferer disclosed IPV	99 (33.1)	188 (46.7)
Learned from relatives/friends	161 (53.8)	113 (28.1)
Learned from a social worker	29 (9.7)	32 (8.0)
Suspected IPV due to sufferer’s behavior/injuries	21 (7.0)	89 (22.1)

*p < 0.001; **More than one response allowed.

Table 2 shows the PHMs’ discussion of IPV with the identified IPV sufferers before and after the intervention. Six months after the intervention, 96.5% of the PHMs discussed IPV with all of the identified sufferers, compared to 67.3% at pre-intervention. Before the intervention, 58.2% of the PHMs suggested that the IPV sufferers should tolerate the violence and be patient; after the intervention, however, only 1.3% of the PHMs made this suggestion. Before the intervention, only 29.4% of the PHMs asked sufferers to seek help from relatives or friends; this increased to 77.1% after the intervention. After the intervention, 22.4% of the PHMs referred sufferers to the Medical Officers of Health; before the intervention, only 6.5% did so. Prior to the intervention, 10.4% of the PHMs suggested that sufferers should avoid conflict situations with abusers and solve problems through friendly discussions; after the intervention, 30.4% made this suggestion. Only 1% of the PHMs acted as mediators and helped couples to solve IPV problems before the intervention. This increased to 15.7% after the intervention.

**Table 2:** **Participants’ pre- and post-intervention practices in discussing intimate partner violence (IPV) with identified sufferers**

**Variable**	**Pre-intervention**	**Post-intervention**
	**n (%)**	**n (%)**
**Discussed the experience of IPV with all identified IPV sufferers** ^*^		
Yes	201 (67.3)	387 (96.5)
No	98 (32.7)	15 (3.5)
**Solutions suggested after discussing IPV** ^**^		
Advised sufferers to be patient and tolerant with the perpetrator	117 (58.2)	5 (1.3)
Asked sufferers to seek help from family/friends	59 (29.4)	299 (77.1)
Asked sufferers to report violence to the police	34 (16.9)	29 (7.5)
Helped sufferers report violence to the police	3 (1.5)	0 (0.0)
Referred sufferers to the Medical Officer of Health/IPV services	13 (6.5)	87 (22.4)
Suggested sufferers improve communication with partners	21 (10.4)	118 (30.4)
Acted as mediators and helped sufferers solve problems with their partners	2 (1.0)	61 (15.7)
**If IPV was not discussed, the reason was…** ^**^		
I believed it was a personal matter	36 (36.7)	0 (0.0)
I thought I might humiliate the sufferer	22 (22.4)	0 (0.0)
I thought the sufferer would get angry if asked	24 (24.5)	6 (37.5)
The sufferer didn’t like to talk	16 (16.3)	14 (87.5)

*p < 0.01; **More than one response allowed.

Before the intervention, 46.8% of the PHMs followed up at least one IPV sufferer after discussing IPV; none followed up all of the sufferers with whom they discussed IPV. Six months after the intervention, 89.7% of the PHMs followed up at least one IPV sufferer after discussing IPV, and 24.5% followed up all of the IPV sufferers.

### Pre- and post-intervention differences in PHMs’ IPV knowledge, perceived barriers, responsibility, and self-confidence

Table 3 shows a comparison of the PHMs’ pre- and post-intervention median total IPV knowledge scores and the median total scores for the following IPV knowledge domains: acts of IPV, health effects of IPV, and laws against IPV. After the intervention, the PHMs’ median total knowledge scores for the acts of IPV increased significantly from 0.83 to 1.00 (*p* < 0.001). The median total knowledge scores for the health effects of IPV also increased significantly from 0.50 to 1.00 (*p* < 0.001). The median total knowledge scores for the IPV laws increased significantly from 0.50 to 0.67 (*p* < 0.001). Additional file 2 shows the PHMs’ responses to individual items in the IPV knowledge domain before and after the intervention. After the intervention, a significantly higher number of PHMs correctly answered IPV knowledge items. For example, PHMs who correctly answered the item “urinary tract infections could be a health effect of IPV” increased from 21.8% pre-intervention to 67.6% post-intervention.

**Table 3:** **Comparison of participants’ pre- and post-intervention intimate partner violence (IPV) knowledge scores**

**Variable**	**Pre-intervention**		**Post-intervention**		**z-score**	***p*** **-value**
	**Median**	**IQR**	**Median**	**IQR**		
**Total knowledge scores**						
Acts of IPV^*^	0.83	0.67–1.00	1.00	1.00–1.00	−11.55	<0.001
Health effects of IPV^*^	0.50	0.25–0.75	1.00	0.75–1.00	−15.09	<0.001
Laws against IPV^*^	0.50	0.33–0.50	0.67	0.67–0.83	−16.41	<0.001
Combined IPV knowledge score^*^	0.62	0.43–0.81	0.88	0.82–0.94	−17.35	<0.001

^*^A detailed comparison of the itemized knowledge scores is provided in Table A2. IQR: interquartile range.

Table 4 shows the comparison of PHMs’ pre- and post-intervention perceived barrier scores. After the intervention, the median total perceived barrier scores decreased significantly from 2.43 to 1.14 (*p* < 0.001). The individual item scores for barriers also decreased significantly from baseline.

**Table 4:** **Comparison of participants’ pre- and post-intervention perceived barrier scores to identify and assist intimate partner violence (IPV) sufferers**

**Variable**	**Pre-intervention**		**Post-intervention**		**z-score**	***p*** **-value**
	**Median**	**IQR**	**Median**	**IQR**		
My workload is too heavy. I do not have enough time to ask about IPV	2.00	1.00–2.00	1.00	1.00–1.00	−14.76	<0.001
I am afraid I will offend the person if I ask about IPV	2.00	2.00–3.00	2.00	1.00–2.00	−10.14	<0.01
It is difficult to get the person alone to ask about violence	2.00	2.00–3.00	1.00	1.00–2.00	−14.75	<0.001
I do not have any training to identify or help those who experience IPV	5.00	3.00–5.00	1.00	1.00–1.00	−17.80	<0.001
Even though I identify IPV, there are no supportive services for sufferers	2.00	2.00–3.00	1.00	1.00–1.00	−14.76	<0.001
I don’t feel like I can help a person who is in a violent relationship	2.00	2.00–3.00	1.00	1.00–1.00	−15.29	<0.001
I am more interested in dealing with my patients’ medical problems	2.00	2.00–3.75	1.00	1.00–1.00	−15.37	<0.01
Total barrier score	2.43	2.14–3.14	1.14	1.14–1.28	−17.52	<0.001

IQR: interquartile range.

Table 5 shows the comparison of the PHMs’ pre- and post-intervention perceived responsibility scores. After the intervention, PHMs’ median total perceived responsibility scores increased significantly from 3.20 to 4.60 (*p* < 0.001). All of the items of the perceived responsibility scale also showed significant increases in scores from baseline.

**Table 5:** **Comparison of participants’ pre- and post-intervention perceived responsibility scores to identify and assist intimate partner violence (IPV) sufferers**

**Variable**	**Pre-intervention**		**Post-intervention**		**z-score**	***p*** **-value**
	**Median**	**IQR**	**Median**	**IQR**		
**The responsibility of a public health midwife includes…**						
Asking about partner violence any time an injury is noticed	3.00	3.00–4.00	5.00	4.00–5.00	−16.62	<0.001
Asking about partner violence any time a serious child injury is noticed	3.00	3.00–4.00	4.00	4.00–5.00	−15.39	<0.001
Listening to an IPV sufferer when violence is disclosed	3.00	3.00–4.00	5.00	5.00–5.00	−15.10	<0.001
Telling a person that a perpetrator’s behavior is not acceptable	3.00	3.00–4.00	4.00	4.00–5.00	−15.64	<0.001
Telling a person that the violence can adversely affect her/his health	3.00	3.00–4.00	5.00	4.00–5.00	−16.40	<0.001
Total responsibility score	3.20	2.80–3.95	4.60	4.20–4.80	−17.30	<0.001

IQR: interquartile range.

Table 6 shows the comparison of PHMs’ pre- and post-intervention self-confidence scores. After the intervention, PHMs’ median total self-confidence scores increased significantly from 1.81 to 2.75 (*p* < 0.001). The scores of all items on the self-confidence scale also showed significant increases from baseline.

**Table 6:** **Comparison of participants’ pre- and post-intervention self-confidence scores to identify and assist intimate partner violence (IPV) sufferers**

**Variable**	**Pre-intervention**		**Post-intervention**		**z-score**	***p*** **-value**
	**Median**	**IQR**	**Median**	**IQR**		
**The current level of self-confidence in…**						
Asking a person whether s/he has experienced IPV	2.00	1.00–2.00	3.00	3.00–3.00	−17.49	<0.001
Taking a sexual history and history of sexual violence	1.00	1.00–2.00	2.00	2.00–3.00	−16.87	<0.001
Knowing what to do if a person says s/he has experienced IPV	2.00	1.00–2.00	3.00	2.00–3.00	−16.66	<0.001
Knowing what to do if a person breaks down and cries	2.00	1.00–2.00	3.00	3.00–3.00	−16.96	<0.001
Assessing the safety of a person experiencing IPV	2.00	1.00–2.00	3.00	2.00–3.00	−19.84	<0.001
Knowing what to do if victim does not want to leave the perpetrator	2.00	2.00–2.00	3.00	3.00–3.00	−16.01	<0.001
Making a referral for a person who has experienced IPV	2.00	2.00–3.00	3.00	3.00–3.00	−14.73	<0.001
Knowing what to do when child violence is co-existing	2.00	1.00–2.00	3.00	2.00–3.00	−15.33	<0.001
Total self-confidence score	1.81	1.38–2.12	2.75	2.62–2.88	−17.43	<0.001

IQR: interquartile range.

## Discussion

The four-day IPV training program for PHMs significantly improved Kandy district PHMs’ ability to identify IPV, discuss IPV experiences with sufferers, and conduct follow-up. It also improved PHMs’ IPV knowledge, reduced their perceived barriers, and improved their responsibility and self-confidence to identify and assist IPV sufferers. The improvements in PHMs’ IPV knowledge and skills after the training might have improved their IPV practices, responsibility, and self-confidence while reducing their perceived barriers.

This program is unique because it improved all aspects of health providers’ IPV practices, knowledge, self-confidence, responsibility, and barrier reduction; previous IPV and/or DV training programs did not show such an improvement across all of these domains. For example, a 2004 US Continuing Medical Education program improved health workers (n = 284) knowledge, attitudes, empathy, and self-reported assessment behaviors about DV, but did not improve their perceived responsibility to counsel DV sufferers [8]. In 2004, another US online IPV training program for community practice physicians could significantly improve their IPV-related attitudes, beliefs, and self-reported practices, although IPV knowledge increased only marginally (*p* = 0.06) [11]. A 2010 two-day intensive IPV training program for Greek general practitioners improved participants’ perceived preparedness and knowledge about IPV, but did not improve their self-reported detection of IPV sufferers [9].

The success of Sri Lanka’s IPV training may be based on three factors. The first factor might be the length of the training (four days), and the time allocated to role-playing and case reports. Previous IPV training programs were conducted for less than two days. Only a few programs used role-playing or case reports to improve participants’ practical skills. According to a study conducted with a group of medical students at the University of California, participants’ opportunities to practice skills and receive feedback can significantly improve the outcome of DV training programs [29]. Second, the program’s culturally sensitive approach might have positively affected its outcome. The PHMs were advised to respect cultural values, encourage harmony between the couple, and act cautiously if suggesting separation from a violent partner. As many Sri Lankan wives are economically dependent on their husbands [16], the PHMs’ interventions to assist IPV sufferers should not compromise the sufferer and/or her children’s safety or wellbeing. Research indicates that culturally sensitive approaches are more effective than other approaches to address IPV [4,24,25]. Third, as PHMs are community health care providers, their experience also may have improved the program’s outcome. Health providers in clinical settings tend to have heavy workloads, which means that they might have little time to discuss IPV with patients and develop a close relationship with sufferers. PHMs, on the other hand, work in the field and have more time with sufferers [18,19], making it easier for them to inquire about IPV.

Importantly, after the training, the majority of the PHMs (46.7%) identified IPV sufferers based on the women’s disclosures. A study conducted with 79 midwives in Bristol in the UK also reported that, after receiving training on DV, midwives could identify a significantly higher number of DV sufferers based on the sufferers’ disclosures [30]; after the training, the midwives routinely inquired about DV. The Sri Lankan PHMs also might have asked more frequently about IPV after their training. This might have improved women’s likelihood of disclosing IPV to PHMs. The sufferers’ relatives or friends also were an important source of information, both before and after the intervention. In Sri Lanka, many women consider experiencing IPV as shameful [16]. In such instances, women might not disclose IPV. However, because the PHMs provide health services for those women, the sufferers’ relatives or friends might disclose IPV. Although this is an indirect disclosure, the PHMs might encourage this kind of IPV disclosure because, ultimately, such disclosures might permit the PHMs to help the sufferers. After the training, only 22% of the PHMs identified IPV sufferers based on external signs, perhaps because the majority of IPV sufferers did not have external injuries or other obvious symptoms, such as features of severe depression or anxiety [1,3].

After discussing IPV with sufferers, PHMs suggested different solutions to prevent further IPV. Before the training, 58.2% of the PHMs suggested that the sufferers tolerate the violence; this might be because many Sri Lankans accept IPV as a normal part of a marital relationship and a wife’s typical situation [16]. However, this practice could aggravate the frequency and severity of violence. After the training, only 1.3% of the PHMs asked the sufferers to tolerate the violence, indicating that PHMs well understood how to manage IPV. The remaining PHMs suggested more effective interventions for the sufferers to prevent further IPV. The majority suggested that the sufferers should seek help from their relatives or friends; this is because abusers become reluctant to hurt sufferers when they have support from others [4,6]. Some PHMs acted as mediators between the couples and helped them to solve their problems. Some others suggested that when a problem arises, the sufferers should avoid conflict situations and solve their problems by friendly communications when husbands are in better moods.

As shown in our results, before the intervention, 16.9% of the PHMs suggested the sufferers should report IPV to the police; after the intervention, only 7.5% made this suggestion. This decrease might be explained by the PHMs’ increased referral of sufferers to the Medical Officers of Health after the intervention. Only 6.5% of the PHMs referred the sufferers to the Medical Officers of Health before the intervention, and this percentage increased to 22.4% after the intervention. Medical Officers of Health provided psychological assistance to sufferers and referred them to legal services when necessary.

Although generalization of our results is limited, similar training might improve PHMs’ IPV practices in other districts of Sri Lanka for two reasons. First, all Sri Lankan PHMs are recruited by the MOH using the same recruitment criteria, receive the same midwifery training, and have the same job descriptions [18,19]. Second, although some socio-cultural differences might exist between Kandy and other districts in Sri Lanka, these differences are likely minimal because Sri Lanka is a small island with only 20 million inhabitants [23].

A program limitation was that it did not require PHMs to document their IPV practices. Because PHMs already complete significant amounts of paperwork reporting on their contraceptive services, immunization services, and clinic services [18,19,22], the MOH did not wish to add more paperwork to PHMs’ duties. However, this might compromise program effectiveness and make it difficult to evaluate the program via document review [7-12]. To improve this situation, the MOH might consider requesting PHMs to provide brief reports of their IPV services by introducing a specific reporting format.

The PHMs’ field handbook provided useful practical information on how to identify and assist IPV sufferers. However, the book’s excessive detail and length (52 pages) might make it less readable for PHMs. The MOH might consider revising the field handbook by removing excessive details. It would be appropriate to keep only practically important information for providing IPV services.

This study has four limitations. First, we did not have a control group to compare with the intervention group. A suitable control group could have been drawn from the two districts adjacent to Kandy (Matale and Nuwaraeliya) [22,23]. However, PHMs in Kandy could meet PHMs in the other two districts and share their new IPV knowledge, which might have led to information contamination, thereby producing inaccurate results [31,32]. Second, we used self-reports to examine the PHMs’ IPV practices and did not directly observe them. This was due to the large geographic distribution of the PHM areas and poor road conditions [23]. Further, such a follow-up might have breached the confidentiality of the IPV information disclosed by IPV sufferers. Third, we did not use a previously validated questionnaire for data collection. This is because there were no previous IPV studies with Sri Lankan PHMs. However, we adopted IPV questions from previously validated scales in other settings [7-12], translated them carefully, and pretested them to confirm their reliability and validity in the Sri Lankan context. Fourth, we followed PHMs for only six months. Several previous studies have also used six-month follow-up periods [8,10,29,30]. Because we evaluated the efficacy of a pilot IPV training program, a longer follow-up period might have delayed the nationwide program implementation.

This study also has several strengths. First, most previous studies on this topic used small sample sizes [7-12]. We included all the PHMs in Kandy district to increase the sample size. Second, in this study, dropout rate was minimal. Previous studies had high dropout rates [30,33]. We avoided postal surveys and used PHMs’ monthly meetings to minimize the dropout rate. Third, we evaluated a training that was designed and conducted to meet international standards [24-26], which improved the quality of data reported in this study.

## Conclusions

The IPV training program for PHMs improved their IPV practices, IPV knowledge, perceived responsibility, and self-confidence in identifying and assisting IPV sufferers while reducing their perceived barriers. The comprehensive and culturally sensitive training program, skill development by role-playing, and the field handbooks may have all been important factors that made this program effective. This training program has the potential to improve PHMs’ skills in preventing IPV and supporting sufferers in other regions of Sri Lanka. Other developing countries might benefit from Sri Lanka’s IPV training and train their community health providers in similar ways.

## Additional files

Additional file 1:
**The correlation between PHMs’ IPV knowledge, perceived barriers, perceived responsibility, and self-confidence scores in the pre- and post-intervention surveys.** Description of data: Shows the correlation of the three scales used in the study with IPV knowledge and among each other. Additional file 2:
**Detailed analysis of PHMs’ pre- and post-intervention IPV knowledge.** Description of data: Shows in detail the comparison of PHMs’ responses to the IPV knowledge items before and after the intervention.
