# Supplementary material for: Training Sri Lankan public health midwives on intimate partner violence: a pre- and post-intervention study
Source: BMC Public Health. 2015 Apr 7;15:331. doi: 10.1186/s12889-015-1674-9 (PMC4394591; doi:10.1186/s12889-015-1674-9)
Supplement: Additional file 1: — The correlation between PHMs’ IPV knowledge, perceived barriers, perceived responsibility, and self-confidence scores in the pre- and post-intervention surveys. Description of data: Shows the correlation of the three scales used in the study with IPV knowledge and among each other. [file 12889_2015_1674_MOESM1_ESM.docx]

**The correlation between PHMs’ IPV knowledge, perceived barriers, perceived responsibility, and perceived confidence scores in the pre- and post-intervention surveys.**

| **Item** | **Perceived barriers** | **IPV knowledge** | **Perceived responsibility** | **Perceived confidence** |
| --- | --- | --- | --- | --- |
| **Pre-intervention** | | | | |
| Perceived barriers | 1.00 | -0.67^*^ | -0.69^*^ | -0.70^*^ |
| IPV knowledge | -0.67^*^ | 1.00 | 0.61^*^ | 0.58^*^ |
| Perceived responsibility | -0.69^*^ | 0.61^*^ | 1.00 | 0.71^*^ |
| Perceived confidence | -0.70^*^ | 0.58^*^ | 0.71^*^ | 1.00 |
| **Post-intervention** | | | | |
| Perceived barriers | 1.00 | -0.24^*^ | -0.28^*^ | -0.35^*^ |
| IPV Knowledge | -0.24^*^ | 1.00 | 0.25^*^ | 0.26^*^ |
| Perceived responsibility | -0.28^*^ | 0.25^*^ | 1.00 | 0.40^*^ |
| Perceived confidence | -0.35^*^ | 0.26^*^ | 0.40^*^ | 1.00 |

^*^Correlation is significant at *p* < 0.01.
