# Supplementary material for: Training Sri Lankan public health midwives on intimate partner violence: a pre- and post-intervention study
Source: BMC Public Health. 2015 Apr 7;15:331. doi: 10.1186/s12889-015-1674-9 (PMC4394591; doi:10.1186/s12889-015-1674-9)
Supplement: Additional file 2: — Detailed analysis of PHMs’ pre- and post-intervention IPV knowledge. Description of data: Shows in detail the comparison of PHMs’ responses to the IPV knowledge items before and after the intervention. [file 12889_2015_1674_MOESM2_ESM.docx]

**Detailed analysis of PHMs’ pre- and post-intervention IPV knowledge.**

| **Variable** | | **Pre-intervention** | **Post-intervention** | ***p*-value** |
| --- | --- | --- | --- | --- |
|  |  | **n (%)** | **n (%)** |  |
| **An act of IPV could be…** | | | | |
| Pushing an intimate partner | Yes^#^ | 367 (89.95) | 407 (99.75) | < 0.01 |
|  | No | 41 (10.05) | 1 (0.25) | < 0.01 |
| Trying to keep an intimate partner from seeing friends | Yes^#^ | 346 (84.80) | 408 (100.0) | < 0.01 |
|  | No | 62 (15.20) | 0 (0.0) | < 0.01 |
| Not giving an intimate partner money when needed | Yes^#^ | 335 (82.11) | 399 (97.79) | < 0.01 |
|  | No | 73 (17.89) | 9 (2.21) | < 0.01 |
| Suspecting an intimate partner for no reason | Yes^#^ | 339 (83.09) | 396 (97.06) | < 0.01 |
|  | No | 69 (16.91) | 12 (2.94) | < 0.01 |
| Demanding an intimate partner to seek permission before doing something | Yes^#^ | 291 (71.32) | 337 (82.60) | < 0.01 |
|  | No | 117 (28.68) | 71 (17.40) | < 0.01 |
| Persuading an intimate partner to have sexual intercourse | Yes^#^ | 344 (84.31) | 399 (97.79) | < 0.01 |
|  | No | 64 (15.69) | 9 (2.21) | < 0.01 |
| **Health effects of IPV could be…** | | | | |
| Low self-esteem | Yes^#^ | 290 (71.08) | 389 (95.34) | < 0.01 |
|  | No | 118 (28.92) | 19 (4.66) | < 0.01 |
| Contusions in the thighs | Yes^#^ | 252 (61.76) | 361 (88.48) | < 0.01 |
|  | No | 156 (38.24) | 47 (11.52) | < 0.01 |
| Visiting doctors with multiple somatic complaints | Yes^#^ | 231 (56.62) | 364 (89.22) | < 0.01 |
|  | No | 177 (43.38) | 44 (10.78) | < 0.01 |
| Urinary tract infections | Yes^#^ | 89 (21.81) | 276 (67.65) | < 0.01 |
|  | No | 319 (78.19) | 132 (32.35) | < 0.01 |
| **IPV laws** | | | | |
| In Sri Lanka, a person can act in courts against psychological abuse by an intimate partner | Yes^#^ | 192 (47.06) | 352 (86.27) | < 0.01 |
|  | No | 216 (52.94) | 56 (13.73) | < 0.01 |
| In Sri Lanka, a person can act in courts against sexual abuse by an intimate partner | Yes | 336 (82.35) | 236 (57.84) | < 0.01 |
|  | No^#^ | 72 (17.65) | 172 (42.16) | < 0.01 |
| A person should always report to the police before filing an IPV case in courts | Yes | 368 (90.20) | 194 (47.55) | < 0.01 |
|  | No^#^ | 40 (9.80) | 214 (52.45) | < 0.01 |
| In most police stations, there is a specific place to receive IPV complaints | Yes^#^ | 291 (71.32) | 382 (93.63) | < 0.01 |
|  | No | 117 (28.68) | 26 (6.37) | < 0.01 |
| For IPV, the Magistrate court can issue a protection order within two weeks | Yes^#^ | 58 (14.22) | 183 (44.85) | < 0.01 |
|  | No | 350 (85.78) | 225 (55.15) | < 0.01 |
| In Sri Lanka, there are supportive services for IPV victims | Yes^#^ | 330 (80.88) | 404 (99.01) | < 0.01 |
|  | No | 78 (19.12) | 4 (0.99) | < 0.01 |

IPV: intimate partner violence; ^#^Correct answer choice
